# To Inventors of Dental Instruments, Chairs, &c.

**Published:** 1851-01

**Authors:** 


*To Inventors of Dental Instruments, Chairs, &c.*—We have received the intimation, from many gentlemen in the profession, of their having made different improvements upon instruments, chairs, and other mechanical appurtenances, seeking our opinion upon their various merits and probable adoption. To all such we would say, that by sending on accurately written descriptions, we would be happy to give them a suitable place in the Journal, thus giving the profession generally an opportunity of testing their merits, and judging of their value.

